# A Microarray Screening Platform with an Experimental Conditions Gradient Generator for the High-Throughput Synthesis of Micro/Nanosized Calcium Phosphates

**DOI:** 10.3390/ijms21113939

**Published:** 2020-05-30

**Authors:** Xiaoyu Li, Zhifeng Shi, Lei Liu, Guanglin Zhu, Jianhua Zhou, Xuetao Shi, Yingjun Wang

**Affiliations:** 1 School of Materials Science and Engineering, South China University of Technology, Guangzhou 510640, China; joylixiaoyu1992@163.com (X.L.); zhfs@scut.edu.cn (Z.S.); 201710102934@mail.scut.edu.cn (L.L.); zglin.520@gmail.com (G.Z.); 2 National Engineering Research Center for Tissue Restoration and Reconstruction, South China University of Technology, Guangzhou 510006, China; 3 Key Laboratory of Biomedical Engineering of Guangdong Province, South China University of Technology, Guangzhou 510006, China; 4 Key Laboratory of Biomedical Materials and Engineering of the Ministry of Education, South China University of Technology, Guangzhou 510006, China; 5 Innovation Center for Tissue Restoration and Reconstruction, South China University of Technology, Guangzhou 510006, China; 6 School of Biomedical Engineering, Sun Yat-sen University, Guangzhou 510275, China

**Keywords:** microfluidics, high-throughput, concentration gradient, calcium phosphates

## Abstract

Calcium phosphates (CaP) represent an impressive kind of biomedical material due to their excellent biocompatibility, bioactivity, and biodegradability. Their morphology and structure highly influence their properties and applications. Whilst great progress has been made in research on biomedical materials, there is still a need to develop a method that can rapidly synthesize and screen micro/nanosized biomedical materials. Here, we utilized a microarray screening platform that could provide the high-throughput synthesis of biomedical materials and screen the vital reaction conditions. With this screening platform, 9 × 9 sets of parallel experiments could be conducted simultaneously with one- or two-dimensions of key reaction condition gradients. We used this platform to establish a one-dimensional gradient of the pH and citrate concentration and a two-dimensional gradient of both the Ca/P ratio and pH to synthesize CaP particles with various morphologies. This screening platform also shows the potential to be extended to other reaction systems for rapid high-throughput screening.

## 1. Introduction

Calcium phosphates (CaP) are of special interest in tissue engineering because they are similar to the major inorganic component of natural hard tissues, such as bones and teeth, and their excellent bioactivity, biocompatibility, and osteoconductivity make them promising biomedical materials [1]. They have been widely studied for bone regenerative applications, such as bone cements, scaffolds, implants, and alloy coatings [2]. For instance, Hesaraki; et al. developed CaP cement with an improved injectability for application in minimally invasive bone defect repair surgery [3]. The morphology and structure of micro/nanosized biomedical materials also play a crucial role in their properties and applications [4,5]. Research has shown that a flake-like CaP coating on an alloy is beneficial for the improvement of osseointegration [6], and hydroxyapatite (HAp) nanorods can self-assemble to enamel-like structures [7] and sphere nano alginate/HAp that could promote bone mineral deposition [8]. However, most studies that have been reported have been based on the traditional trial and error method, namely the use of existing theoretical and empirical knowledge on materials, by adjusting the research material ratio, changing the reaction conditions, and performing characterization tests and inspections, in order to eventually obtain the required materials [9]. Such research methods usually require a long research and development cycle and are accompanied by a waste of resources, so a better and more effective research mode is desired [10,11].

With the continuous development of microfluidic technology and its broad application in high-throughput experiments [12,13], this technique has allowed people to perform traditional laboratory experimental procedures, such as sample pretreatment, mixing, reaction, extraction, and separation, on one chip [14,15,16]. For example, Liu; et al. designed a multiplexed microfluidic platform which could high-throughput fabricate core–shell structured nanocomposites, and the prepared core–shell nanocomposites exhibited ultra-high drug loading and controlled drug release from polymer-based nanoparticles [17]. Liu; et al. presented a microfluidic nanoprecipitation platform which could produce diverse types of homogeneous nanoparticles with the desirable nanomaterial production rate for clinical studies [18]. Additionally, in our previous work, we designed a microfluidic platform with 6 × 6 microreactors, and a series of CaP with various morphologies were fabricated by high-throughput screening of the reactant concentration gradient manipulated through volume control [19].

Based on the above background, this study utilized a microarray screening platform to screen the effects of the reactant concentration, additive concentration, and pH on the micro/nanosized CaP particle morphology. Compared with our previous work, the experiment throughput was increased to 9 × 9 and the concentration gradients were generated by reagent solution diffusion in hydrogel. Through establishing a one-dimensional gradient of pH and trisodium citrate dihydrate (Na_3_C_6_H_5_O_7_·2H_2_O, Na_3_Cit) concentration separately and a two-dimensional gradient of both the Ca/P molar ratio (Ca/P) and pH, CaP particles with different morphologies were prepared. The experimental results show that the microarray screening platform can realize a controllable chemical substance concentration gradient and pH gradient, and quickly and effectively screen a wide range of experimental conditions in one experiment, providing data support for the formation mechanism of crystals with different morphologies.

## 2. Results

**Figure 1:**
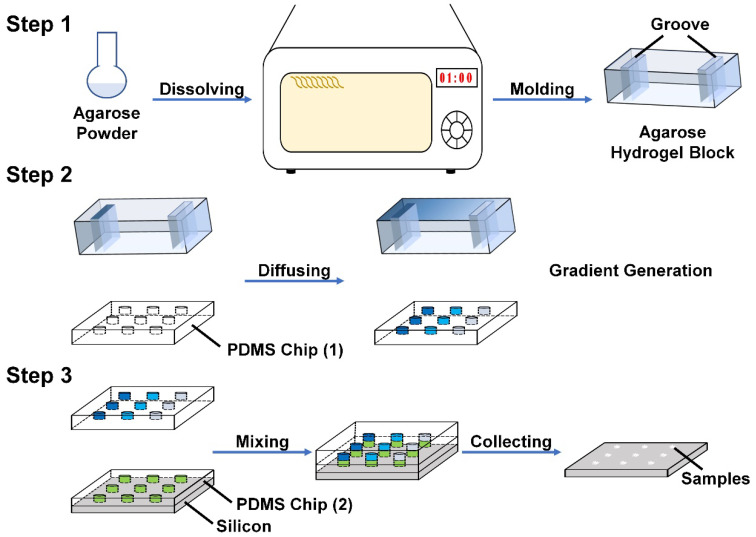
The key steps of performing experiments in the screening platform: Step 1: a hydrogel block with two grooves was fabricated; Step 2: different concentrations of reagent were added into the two grooves respectively, generating a concentration gradient of reagent in the hydrogel block and the half perforated PDMS chip (1) underneath due to diffusion; and Step 3: the reaction was triggered in the microreactors formed by matched arrayed holes in the PDMS chip (1) and fully perforated PDMS chip (2), which was placed on a silicon wafer containing solution with all other required reagents, and precipitated samples of the array were left on the silicon wafer after the reaction.

Figure 1 shows the key steps in the screening experiment. Briefly, by forming a hydrogel block with two grooves (Step 1) and adding different concentrations of reagent A into the grooves respectively, reagent A was transported through the hydrogel block due to the concentration difference and a concentration gradient of reagent A was thus generated in the hydrogel block and in the half perforated polydimethylsiloxane (PDMS) chip (1), which was placed beneath the hydrogel block (Step 2). By adding a solution with all other required reagents to the fully perforated PDMS chip (2), which was placed on a silicon wafer, the reaction was immediately triggered when the solutions in the two PDMS chips were brought into contact with each other. Since both PDMS chips were fabricated with the same polymethyl methacrylate (PMMA) mold, the holes in the different chips could be matched perfectly and thereby formed an array of sealed microreactors. After removing the PDMS chips, the products were deposited in the same position array on the silicon wafer (Step 3). The photographs of the PMMA mold and both PDMS chips are displayed in Figure S1 in the supplementary materials, and the procedures employed for fabricating the two PDMS chips are shown in Figure S2 in the supplementary materials and described in detail in the Materials and Methods part.

As a demonstration, we used a simple red ink experiment conducted following the steps presented in Figure 1 to prove that a stable concentration gradient could be generated through this platform. As shown in Figure S3C in the supplementary materials, by adding red ink in the left groove of the hydrogel block and adding deionized water in the right groove while a PDMS chip (1) was placed under the hydrogel block, after 24 h, the hydrogel block showed a continuous color gradient; from left to right, the color of the hydrogel block gradually changed from dark red to light pink, as well as the cylindrical holes in the square array in the PDMS chip (1).

### 2.1. High-Throughput Screening One-Dimensional Gradient for Calcium Phosphate Synthesis

**Figure 2:**
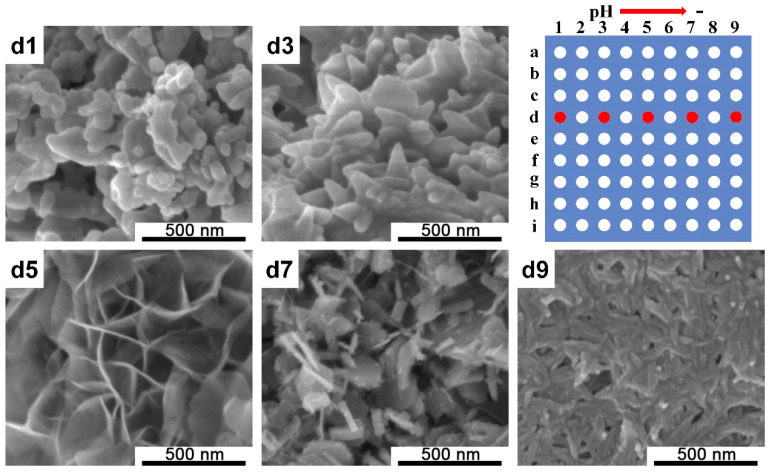
SEM images of calcium phosphate (CaP) structures observed in the screening platform and a schematic of the microreactors array. From d1 to d9, the pH values varied from 13 to 7. The red spots correspond to the positions of the precipitated samples of SEM acquisition.

By placing the PDMS chip (1) containing 0.12 M ammonium phosphate dibasic [(NH_4_)_2_HPO_4_] and 0.50 M Na_3_Cit with a pH gradient from 13 to 7 across different columns of the holes array on the PDMS chip (2), which was fixed on a silicon wafer that contained 0.20 M calcium nitrate tetrahydrate [Ca(NO_3_)_2_·4(H_2_O)] solution, when the two solutions in different PDMS chips were brought into contact with each other, the reaction was immediately triggered, and calcium phosphates began to be generated inside the microreactors. Figure 2 shows the scanning electron microscope (SEM) images of the generated calcium phosphates’ structure on the silicon wafer and from left to right, the pH value changed from 13 to 7. As can be observed in Figure 2, apparently, the pH value of the reactant solution had a crucial influence on the morphologies of the calcium phosphates formed. When the pH value was relatively low, CaP exhibited a one-dimensional nanorod morphology with a length of about ~150–200 nm and a nanosheet morphology was observed when the pH value was increased, which then changed into a network structure formed by a large piece of thin flakes. Additionally, irregular nanoparticles were formed in a strong alkaline environment. Figure S4 in the supplementary materials shows the SEM images of the calcium phosphate structures generated at a3 and h3, and as can be observed, irregular nanoparticles were formed at a3, d3 (Figure 2), and h3, which proved the stability of the pH gradient in the same column.

**Figure 3:**
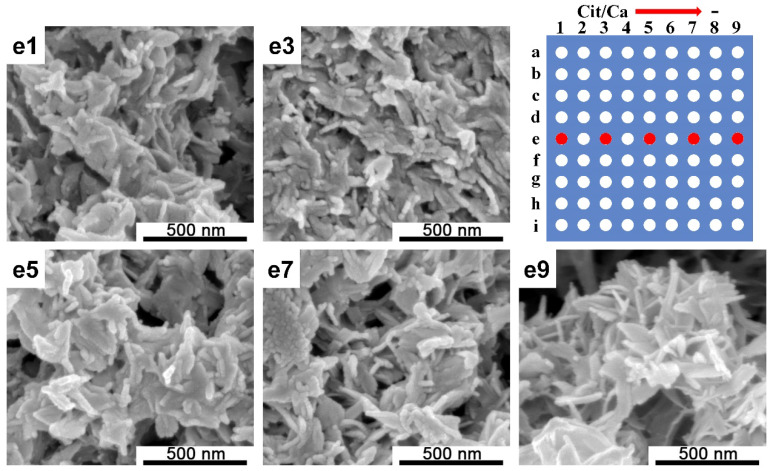
SEM images of CaP structures observed in the screening platform and a schematic of the microreactors array. From e1 to e9, the Cit/Ca ratio varied from 2.5 to 0. The red spots correspond to the positions of the precipitated samples of SEM acquisition.

We also established a Na_3_Cit concentration gradient to explore the additive effect for calcium phosphate synthesis. By mixing the 0.20 M Ca(NO_3_)_2_ solution in the PDMS chip (2) with a mixed solution of 0.12 M (NH_4_)_2_HPO_4_ and the Na_3_Cit concentration gradient (varied from 0.5 to 0 M) in the PDMS chip (1), calcium phosphates were generated inside the microreactors and the morphologies of calcium phosphates indicated the influence of Cit^3−^ during crystallization. As can be seen in Figure 3, under mild alkaline conditions, compared to the pH change, Cit/Ca variation (from 0 to 2.5) did not significantly affect the CaP morphologies; as the concentration of citrate increased, the particles transformed from loose thorn-like structures to dense short rod-like structures, and finally, rod-like and sheet-like structures were obtained.

### 2.2. Scale-Up Experiments for Calcium Phosphate Synthesis Based on Screening Results

We conducted scale-up experiments based on the preceding pH screening platform conditions to determine the fidelity and reliability of our screening platform and the reaction volume was increased from V_0_ ≈ 200 nL to V = 1 mL. The scale-up experiments were performed in 1.5 mL EP tubes (eppendorf tubes). Briefly, five groups of mixed solution [0.12 M (NH_4_)_2_HPO_4_ and 0.50 M Na_3_Cit] were prepared and the pH of each solution was adjusted to 7.0, 9.0, 10.0, 11.0, and 13.0, respectively. Then, 0.5 mL of each solution was taken and mixed with 0.5 mL 0.20 M Ca(NO_3_)_2_ solution separately. During the experiment, it was found that for neutral and weakly alkaline environments, the reactant solutions were relatively clear at the beginning after mixing; when the pH ≥ 10, each group of reactant solutions began to react immediately after mixing, and the reaction solutions were turbid. However, after 24 h of reaction, more products were obtained from pH = 10 to a neutral environment, and the yield increased significantly with the decrease of the pH value. We could not collect enough products for characterization when the pH of the mixed solution was adjusted to 11.0; however, when the pH increased to 13, the yield of CaP increased again. The collected precipitates were washed, dried, and preserved for SEM, Fourier transform-infrared (FT-IR), and X-ray diffraction (XRD) characterization.

**Figure 4:**
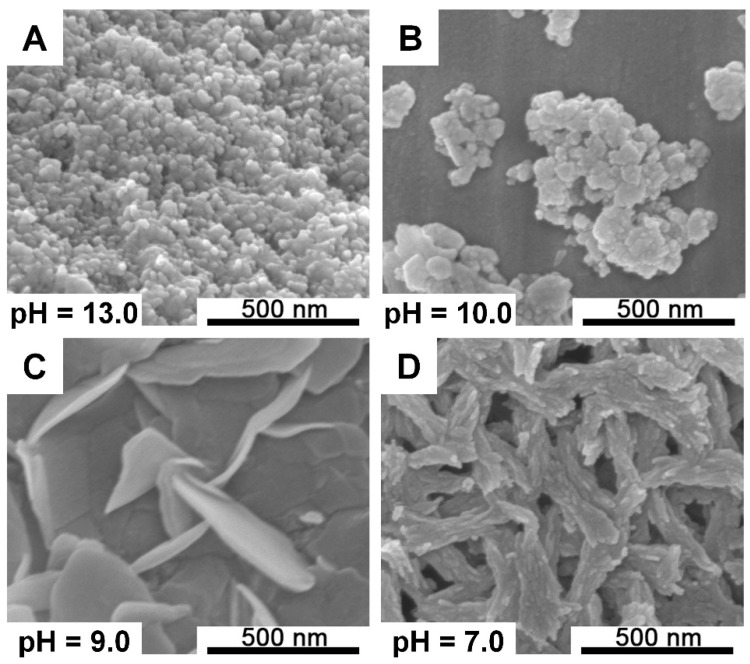
SEM images of CaP structures observed in scale-up experiments. (**A**) pH = 13.0; (**B**) pH = 10.0; (**C**) pH = 9.0; (**D**) pH = 7.0.

It can be seen from Figure 4 that in a neutral environment, a short nanorod-shaped structure was obtained with the length of approximately 500 nm and the two ends of the rod were not well-developed, indicating that the crystals showed a poor crystallinity. When the pH of the mixed solution was adjusted to 9, CaP exhibited a network structure formed by broad flakes. Additionally, the size of the crystals shrank significantly in a strong alkaline environment, and irregular nanoparticles were formed. Compared with the microfluidic screening platform results, we found that the morphologies of the CaP formed exhibited similar trends, although the details were slightly different, which proved that the presented screening platform was accurate and reliable and displayed practicality.

Figure S5 in the supplementary materials shows the FT-IR and XRD results of CaP generated in the scale-up experiments. From the FT-IR absorption spectra of all prepared samples, the characteristic absorption peaks of calcium phosphate at 1033, 602, and 565 cm^−1^ were observed. Moreover, the XRD patterns indicated that the main phase of prepared CaP was the apatite phase, as the main diffraction peaks correspond to the characteristic diffraction peaks of apatite on the standard PDF card. When comparing all XRD patterns, relatively strong and characteristic peaks were observed in the neutral environment and strongly alkaline environment; when the pH was adjusted to 7, citrate characteristic diffraction peaks emerged, which was not observed under other conditions.

### 2.3. High-Throughput Screening Two-Dimensional Gradient for Calcium Phosphate Synthesis

To create a two-dimensional gradient in the platform, we also designed another set of polycaprolactone (PCL) molds and hydrogel blocks with four grooves (left, right, top, and bottom) could be fabricated by the molds. Figure S3D in the supplementary materials presents a photograph of the PCL base and the matched PCL frame. The effects of both the Ca/P ratio and pH value were screened using this newly designed platform. NaOH solution with the pH value adjusted to 13 was added in the top groove of the hydrogel block and deionized water was added in the bottom groove, while a 0.06 M Ca(NO_3_)_2_ solution was added in the right groove of the hydrogel block and another 0.24 M Ca(NO_3_)_2_ solution was added in the left groove. After diffusion, the PDMS chip (1) which contained a stable two-dimensional concentration gradient of both Ca^2+^ (decreased from left to right) and the pH value (increased from bottom to top) was flipped over and solutions in the holes array were reacted with 0.12 M (NH_4_)_2_HPO_4_ solution in the PDMS chip (2). The precipitates were preserved for SEM characterization.

**Figure 5:**
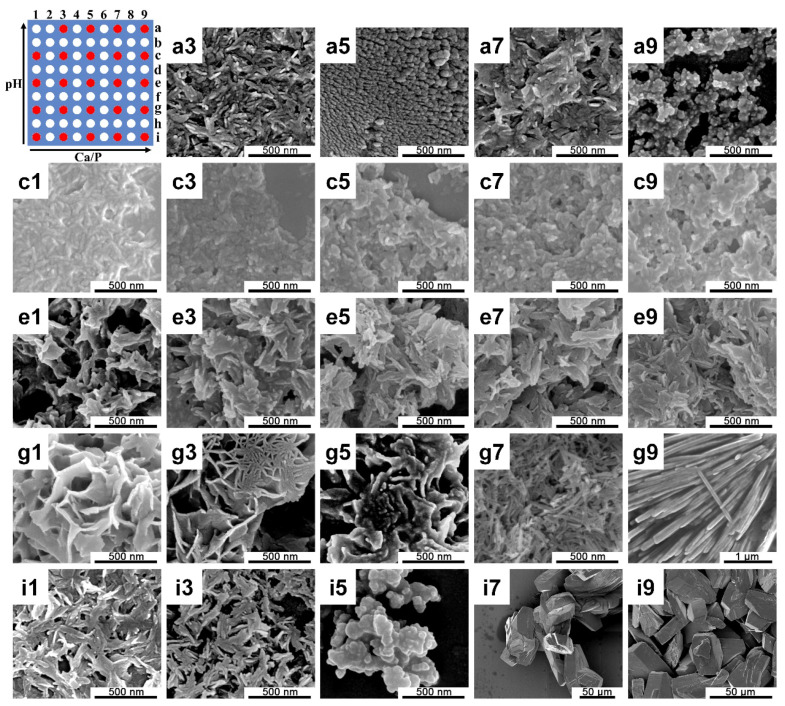
A schematic of the microreactors array and SEM images of CaP structures observed in the screening platform with a two-dimensional gradient for the pH values and the Ca/P ratio, separately. From rows i to a (bottom to top), the pH values increased from 7 to 13; from columns 1 to 9 (left to right), the Ca/P ratio increased from 0.5 to 2.0. The red spots correspond to the positions of the precipitated samples of SEM acquisition.

Figure 5 shows the SEM images of prepared CaP obtained in this screening platform and the changing directions of this two-dimensional gradient of the pH and Ca/P ratio. In neutral environments, a nanorod-like structure was observed at a low Ca/P condition and by increasing the concentration of Ca^2+^, the CaP structure changed to a micron block structure. As for mild alkaline conditions, a sponge structure appeared and gradually transformed into a micron thin strip structure. As the concentration of OH^−^ in the solution further increased, and the change of Ca/P no longer exhibited significant effects on the morphology of CaP, and as the pH of the solution increased, the spiny cluster structure transformed into irregular nano-scale random particles.

## 3. Discussion

In this study, a microarray screening platform with high-throughput synthesis and screening functions was utilized. This screening platform contained three parts: screening chips, which provided microreactors for 9 × 9 parallel experiments; hydrogel blocks with a concentration gradient generation function; and a positioning device. A one-dimensional gradient of the pH and Na_3_Cit and two-dimensional gradient of both the Ca/P ratio and pH were established through this platform, and a series of CaP particles with various morphologies were synthesized. Compared with our previous work, as mentioned earlier, the reactor array was increased from 6 × 6 to 9 × 9, which led to an increase in the experiment throughput; meanwhile, the reaction volumes were decreased to about 200 nL, which helped to save the reagents. More importantly, the concentration gradient generation methods were different. The concentration gradient generation in this platform was driven by diffusion, by adding a high concentration of solution A in the left grooves while adding a low concentration of solution A in the right grooves of the hydrogel block. Reagent A was transported from a high-concentration region to low-concentration region and during this process, a concentration gradient of reagent A could be generated in the hydrogel block. When we placed the PDMS chip (1) beneath the hydrogel block, reagent A also diffused into the corresponding holes in the PDMS chip (1) from the hydrogel block, resulting in a concentration gradient across the hole in the PDMS chip (1). The red ink experiment (Figure S3C in the supplementary materials) demonstrated the diffusion process and confirmed that a stable concentration gradient could be generated through this platform. Meanwhile, the color of the hole solution in the same column was basically the same, which proved that the gradient was only generated in the horizontal direction and the consistency in the vertical direction of diffusion. The similarity of the SEM images of the calcium phosphate structure shown in a3 and h3 (Figure S4 in the supplementary materials) and d3 (Figure 2) also supported this conclusion, as irregular nanoparticles were formed in the same column (column 3), which proved the stability and reproducibility of this screening platform.

In order to validate the practicability of our platform, the morphologies of calcium phosphates were screened utilizing this platform due to their excellent biocompatibility with soft tissues, muscles, and skin [20,21,22], and due to the fact that their morphology and structure highly influence the properties and applications for micro/nano calcium phosphate (CaP) materials [23,24]. The synthesis of CaP was based on a simple wet chemical precipitation method [25] with minor modifications, as described in our previously published article [19]. Through the mixing of Ca(NO_3_)_2_ solution and (NH_4_)_2_HPO_4_ solution, a series of calcium phosphate precipitations with various morphologies could be generated due to the concentration gradient of vital reagent formed in this platform. Meanwhile, to determine the fidelity and reliability of our screening platform, and considering that the pH value of the reactant solution significantly influences the morphologies of the calcium phosphates particles formed (Figure 2), we conducted scale-up experiments based on the preceding pH screening platform conditions. The morphologies of calcium phosphatase generated in the pH screening experiment (Figure 2) and scale-up experiment (Figure 4) exhibited a certain degree of consistency under similar reaction conditions. In a neutral environment, short nanorod-shaped structures were obtained, and a network structure formed by a large piece of thin flakes emerged due to an increased pH value. Additionally, in a strong alkaline environment, the main morphology of CaP was irregular nanoparticles. The morphologies of the CaP formed exhibited similar trends, although the details were slightly different, which proved that the presented screening platform was accurate and reliable and displayed practicality.

The consistency of both FT-IR and XRD results (Figure S5 in the supplementary materials) indicated that the products formed under all reaction conditions in the scale-up experiments were calcium phosphate in the apatite phase. From FT-IR absorption spectra, the characteristic absorption peaks of calcium phosphate at 1033, 602, and 565 cm^−1^ were observed, of which the peak at 1033 cm^−1^ was caused by ${\mathrm{PO}}_{4}^{3-}$ antisymmetric stretching vibration, while the peaks at 602 and 565 cm^−1^ were caused by ${\mathrm{PO}}_{4}^{3-}$ asymmetric change angular vibration [26]. The absorption peak at 1400 cm^−1^ was caused by ${\mathrm{CO}}_{3}^{2-}$ being adsorbed on the surface, while peaks at 3420 and 1600 cm^−1^ were caused by O-H stretching vibration and variable angle vibration in H_2_O [26]. When the pH decreased, the wavenumber decreased, and the characteristic peak appeared to be red-shifted. As the infrared spectrum is a vibration spectrum, the energy required is usually low and a characteristic absorption peak moving to a low wavenumber means that the energy required for vibration becomes lower, and the representative group is more unstable. It is speculated that the increase in pH is more conducive to the stable existence of calcium phosphate, and the resulting product is more stable. As for the XRD results, a broad diffraction peak appeared between 2θ = 31.8 and 33°, caused by overlapped crystal planes (211), (112), and (300). This was observed in all patterns, which suggested that the crystallinity of the apatite formed was relatively low [27]. The characteristic peak at 2θ = 25.9° represented the (002) crystal plane in the c-axis direction [28]. When comparing all XRD patterns, relatively strong characteristic peaks were observed in the neutral environment and strongly alkaline environment; when the pH was adjusted to 7, the observed (200) crystal plane at 5° and (400) crystal plane at 11° suggested the existence of citrate, which was not observed under other conditions and might have been caused by an insufficient centrifugal washing process.

The morphology of the crystal depends on the relative growth rate of the crystal planes. When citrate was involved in the system, Cit^3−^ formed a complex with Ca^2+^, and when the reaction proceeded, Cit^3−^-Ca^2+^ gradually hydrolyzed, releasing Ca^2+^ and controlling the crystal nucleation and growth process. The presence of citrate promoted the growth of crystals along the *c*-axis [29], and a nanorod-like structure was formed in a neutral environment, as shown in d9 (Figure 2). Under mild alkaline conditions, short rod-like structures were transformed into loose thorn-like structures when the concentration of citrate increased (Figure 3). The OH^−^ in the reaction system promoted the growth of crystals along the *a*-axis and *b*-axis [30], and a nanosheet structure was observed, as shown in d5 and d7 (Figure 2); however, when there was an excess of OH^−^ in the solution, the supersaturation of the solution increased, the solution system burst into nucleation and crystallization, the regulating effect of citric acid no longer played a leading role, and there were not enough resources to support the growth of CaP particles. Therefore, irregular nanoparticles were formed, as shown in d1 and d3 (Figure 2). The same situation happened in the two-dimensional gradient screening experiment, as shown in Figure 5 row a to row c.

We have presented a microfluidic screening platform which can simultaneously conduct 9 × 9 sets of parallel experiments and significantly save time and resources for the rapid and high-throughput screening of one-dimensional or two-dimensional key reaction condition gradients and their effects on the product morphology. Using this platform, we screened the influence of the pH gradient and citrate additive concentration gradient on calcium phosphate synthesis, in which the change of pH significantly affected the morphologies of calcium phosphate; meanwhile, we also established a two-dimensional gradient of both Ca/P and pH, and the effect of Ca/P and pH variation on calcium phosphate synthesis was observed. The scale-up experiments proved the practicability and reliability of the design platform. This high-throughput technology can provide a massive amount of data for the establishment of a material database and can also be used as a tool for experimental parameter optimization. In addition, this is a universal screening platform that can be expected to be utilized in other reaction systems for rapid high-throughput screening.

## 4. Materials and Methods

### 4.1. Chemicals and Materials

Agarose was purchased from Biowest, and calcium nitrate tetrahydrate [Ca(NO_3_)_2_·4(H_2_O)], ammonium phosphate dibasic [(NH_4_)_2_HPO_4_], and trisodium citrate dihydrate (Na_3_C_6_H_5_O_7_·2H_2_O, Na_3_Cit) were purchased from Sigma–Aldrich, St. Louis, United States and used as received. Sodium hydroxide (NaOH, 1.0000 M) was purchased from Shanghai Aladdin Bio-Chem Technology Co., LTD, Shanghai, China. Polydimethylsiloxane (PDMS) prepolymer (Dowsil TM 184 silicone elastomer kit) was obtained from Dow Europe GmbH, Wiesbaden, Deutschland. The water used in all reactions was deionized water.

### 4.2. Fabrication of the Screening Chip

The PMMA mold used to manufacture PDMS chips was fabricated following the method presented by Zhou et al. [31]. This PMMA mold contained a 9 × 9 micro holes array, the diameter and depth of the microcolumns were both 500 μm, and the distance between the centers of two adjacent microcolumns was 3 mm. Figure S1A in the Supplementary Materials shows photographs of the PMMA mold and Figure S1B,C in the supplementary materials show three-dimensional model and stereomicroscope images of the selected hole of the PMMA mold, respectively.

The procedure of fabricating the PDMS chip (1) with partially perforated holes and the chip (2) with completely perforated holes is introduced in Figure S2 in the supplementary materials. To fabricate the PDMS mold, the PMMA mold was placed in a clean culture dish with the surface displaying the micro holes array facing up, and the uncured PDMS was prepared by mixing the silicon elastomer base with the curing reagent at a certain ratio (= 10:1 *w*/*w*) and vigorously stirring it to obtain a uniform mixture before vacuuming. After about 45 min of vacuuming to remove the generated air bubbles, the uncured PDMS was poured into the culture dish until it covered approximately 2 mm of the PMMA mold (as shown in Figure S2A in the supplementary materials), and the mixture was vacuumed to remove the residual air bubbles. The culture dish was heated in a drying oven at 65 °C for 2 h to cure the PDMS, and then, the cured PDMS chips were carefully peeled off the PMMA mold. The PDMS mold was then fabricated after cutting off superfluous PDMS. Next, the PDMS chip (1) was fabricated utilizing the PDMS mold (as shown in Figure S2B in the Supplementary Materials) and the procedures were basically the same as those employed to fabricate the PDMS mold. And as for the PDMS chip (2) with fully perforated holes, a silicon wafer (40 × 40 × 0.725 mm^3^) was placed beneath the PDMS mold while the PDMS surface with microcolumns was placed face down and the uncured PDMS covered the PDMS mold (as shown in Figure S2C in the supplementary materials). The mixture was also vacuumed to remove the air between the silicon wafer and the PDMS mold. The PDMS prepolymer was heated in a drying oven at 65 °C for 2 h to cure the PDMS, and then, the cured PDMS chips were carefully peeled off the PDMS mold and preserved in sealed clean culture dishes prior to use. Photographs of the PDMS chip (1) on a PMMA block (4 × 4 × 0.5 cm^3^) and the chip (2) on a silicon wafer are displayed in Figure S1D,E in the Supplementary Materials.

### 4.3. Generation of the Reagent Concentration Gradient

The key aim of this screening platform is to form a reagent concentration gradient inside the holes in PDMS chips. A hydrogel block with two grooves was required and by adding high-concentration solution of reagent A in the left groove and adding low-concentration solution of reagent A in the right groove, reagent A could diffuse through the hydrogel block from left to right and a stable concentration gradient of reagent A could be generated inside the hydrogel block after a sufficient diffusion time, as well as cylindrical holes in the square array in the PDMS chip (1) (as displayed in Figure 1). The procedure of fabricating the hydrogel block is shown in step 1 of Figure 1. The hydrogel block was formed in a PCL frame and a Teflon base (Figure S3B in the supplementary materials), and a 3D modeling diagram of the application of the Teflon base and PCL frame is provided in Figure S3A, where the translucent grey part represents the PCL lid (dimensions of 5.6 × 8.6 × 2.5 cm^3^) and the outframe (inner dimensions of 5 × 8 × 2.5 cm^3^), while the yellow part represents the Teflon base with two rectangular convex plates (dimensions of 40 × 10 × 7 mm^3^) to form the two grooves in the hydrogel block. The distance between the two rectangular convex plates was 30 mm. The agarose powder was dissolved in deionized water by heating it in a microwave oven (Midea, Guangzhou, China) at 700 W for 3 min, which formed a 1.0 wt% agarose solution. Then, the solution was carefully transported to the combined PCL outframe and the Teflon base and was left to solidify when cooling to room temperature, which produced the hydrogel block. The distance between the two grooves (40 × 10 × 7 mm^3^) was 30 mm and the thickness of the hydrogel block was around 10 mm.

As a demonstration, we used a simple red ink experiment to prove that a stable concentration gradient could be generated through this platform. As shown in Figure S3C in the supplementary materials, by adding red ink in the left groove of the hydrogel block and adding deionized water in the right groove while a PDMS chip (1) was placed under the hydrogel block, after 24 h, a continuous color gradient could be generated in the hydrogel block and the cylindrical holes in the square array in the PDMS chip (1).

To address the difficulties of the precise manual alignment of matching holes, a positioning device (Figure S1F in the Supplementary Materials) was designed, which included a PCL base (6 × 6 × 0.5 cm^3^) with a convex plate (4 × 4 × 0.1 cm^3^), an outframe (inner dimensions of 4 × 4 × 1 cm^3^), and a positioner (4 × 4 × 0.5 cm^3^) which had four cylinder protrusions (φ = 500 μm; H = 400 μm) with positions that matched the microcolumns on the PMMA molds at the four corners. The PMMA block or the silicon wafer was placed on the convex plate and the outframe was placed around the convex plate on the PCL base. Meanwhile, the PDMS chip was located on the positioner with the holes at the four corners nested over the four cylinder protrusions respectively and the positioner was pressed through the outframe until the PDMS chip was adhered on the PMMA block or the silicon wafer, as well as the hole array on the PDMS chip, was at a fixed position.

### 4.4. High-Throughput Screening One-Dimensional Gradient for Calcium Phosphate Synthesis

Figure 1 presents the general experimental procedures and screening experiments that were conducted to explore the effect of the pH gradient for the synthesis of calcium phosphates. The PDMS chip (1) which was fixed on a PMMA block through the positioning device was treated with plasma for 3 min and placed under a hydrogel block. Later, a mixed solution of 0.12 M (NH_4_)_2_HPO_4_ and 0.50 M Na_3_Cit (pH was adjusted to 13) was added in the left groove of the hydrogel block, while another mixed solution of 0.12 M (NH_4_)_2_HPO_4_ and 0.50 M Na_3_Cit (pH was adjusted to 7) was added in the right groove, and after 24 h, a mixed solution of 0.12 M (NH_4_)_2_HPO_4_ and 0.50 M Na_3_Cit with a stable pH gradient was generated in the hydrogel block due to diffusion, as well as cylindrical holes in the square array in the PDMS chip (1) beneath the hydrogel block (as shown in Figure 1). On the other hand, 0.20 M Ca(NO_3_)_2_ solution was prepared and added into the holes of the PDMS chip (2) which was fixed on a silicon wafer by the positioning device and a 1 mL syringe was used to remove the air bubbles inside the holes. Additionally, the extra solution on the surface of the PDMS chip (2) was absorbed by filter paper. Subsequently, the PDMS chip (2) fixed on a silicon wafer was placed on the PCL base surrounded by the outframe and the PDMS chip (1) fixed on the PMMA block was flipped over and pressed through the outframe until the PDMS chip (1) came into contact with the surface of the PDMS chip (2). As both of the PDMS chips were fabricated by the same PMMA mold, the holes in the PDMS chip (2) with a uniform Ca(NO_3_)_2_ concentration could be aligned precisely with the holes in the PDMS chip (1) that contained (NH_4_)_2_HPO_4_ and Na_3_Cit solution with a pH gradient. When the two solutions in different PDMS chips were brought into contact with each other, the reaction was immediately triggered, and calcium phosphates began to be generated inside the microreactors. A weight (500 g) was placed on the PMMA block to provide better contact and a seal between the PDMS chips. The whole platform was moved into a shaking table (the temperature was maintained at 37 °C for 24 h, and then transferred into a vacuum drying oven to remove the solvent. Later, it was immersed in deionized water for 3 days to remove soluble impurities. After peeling off the PDMS chips, the generated calcium phosphate structure was left in an array on the silicon wafer for characterization.

We also built a Na_3_Cit concentration gradient to explore the additive effect for calcium phosphate synthesis. A mixed solution of 0.12 M (NH_4_)_2_HPO_4_ and 0.50 M Na_3_Cit was added in the left groove of the hydrogel block, while a 0.12 M (NH_4_)_2_HPO_4_ solution was added in the right groove, and after 24 h, a stable 0.12 M (NH_4_)_2_HPO_4_ solution with an Na_3_Cit concentration gradient (varied from 0.50 M to 0) was generated in the hydrogel block and the holes in the PDMS chip (1) beneath the hydrogel block. Then, the PDMS chip (1) was brought into contact with the PDMS chip (2) which contained 0.20 M Ca(NO_3_)_2_ solution by the positioning device and the reaction was triggered. Except for the solutions added in the grooves of the hydrogel block, the rest of the procedures remained the same as in the previous experiment.

### 4.5. Scale-Up Experiments for Calcium Phosphate Synthesis Based on Screening Results

To determine whether the structures of the samples were the same between the samples generated in this screening platform and general laboratory experiments, we designed scale-up experiments based on the screening experimental conditions. The scale-up experiments were carried out in 1.5 mL EP tubes and the reaction volume was increased from V_0_≈ 200 nL to V = 1 mL. Five groups of mixed solutions (0.12 M (NH_4_)_2_HPO_4_ and 0.50 M Na_3_Cit) were prepared and the pH of each solution was adjusted to 7.0, 9.0, 10.0, 11.0, and 13.0, respectively. Then, 0.5 mL of each solution was taken and added into 1.5 mL EP tubes. Meanwhile, 0.20 M Ca(NO_3_)_2_ solution was prepared and 0.5 mL was placed in the EP tubes with mixed solutions of (NH_4_)_2_HPO_4_ and Na_3_Cit at various pH values. The EP tubes were put into the shaking table (the temperature was maintained at 37 °C for 24 h. After aspirating the supernatant, the obtained white precipitates were washed thoroughly with deionized water and placed on filter paper for air drying. The collected precipitates were preserved for characterization.

### 4.6. High-Throughput Screening Two-Dimensional Gradient for Calcium Phosphate Synthesis

To generate a two-dimensional gradient in the platform, we designed another set of molds which could generate a hydrogel block with four grooves (left, right, top, and bottom). The PCL base and the matched PCL frame (inner dimensions of 8 × 8 × 2.5 cm^3^) are shown in Figure S3D in the supplementary materials. The newly designed PCL base contained four rectangular convex plates (dimensions of 28 × 10 × 7 mm^3^) and the distance between the two opposite rectangular convex plates was 35 mm. The procedure of fabricating a hydrogel block with four grooves was basically the same as shown in step 1 of Figure 1. By adding a high-concentration solution of reagent A in the left groove and adding a low-concentration solution of reagent A in the right groove, while adding a high-concentration solution of reagent B in the top groove and adding a low-concentration solution of reagent B in the bottom groove, reagent A could diffuse through the hydrogel block from left to right and reagent B could also diffuse through the hydrogel block from top to bottom. Moreover, a stable two-dimensional concentration gradient of both reagent A and B could be generated inside the hydrogel block and the cylindrical holes in the square array in the PDMS chip (1) after enough diffusion time.

We screened the influence of the Ca/P and pH value on the morphologies of synthesized calcium phosphates using this platform. NaOH solution with a pH value adjusted to 13 was prepared and added in the top groove of the hydrogel block and deionized water was added in the bottom groove, while a 0.06 M Ca(NO_3_)_2_ solution was added in the right groove of the hydrogel block and another 0.24 M Ca(NO_3_)_2_ solution was added in the left groove. After 24 h, a stable two-dimensional concentration gradient of both Ca^2+^ (decreased from left to right) and the pH value (increased from bottom to top) could be generated inside the hydrogel block and the holes array in the PDMS chip (1). Then, the PDMS chip (1) was flipped over and brought into contact with the PDMS chip (2) which contained 0.12 M (NH_4_)_2_HPO_4_ solution by the positioning device and the reaction was triggered. Except for the solutions added in the grooves of the hydrogel block, the rest of the procedures remained the same as in the previous experiment.

### 4.7. Characterization

SEM imaging was conducted utilizing a MERLIN FE-SEM (field emission scanning electron microscope) instrument from Carl Zeiss, Oberkochen, Germany after the samples were sprayed with Pt for 60 s. The structure of the sample was characterized by XRD analysis using an Empyrean (PANalytical B. V, Almelo, The Netherlands) instrument with Cu Kα radiation (λ = 1.540598 nm). The FT-IR spectra were measured in KBr on a CCR-1 (Thermo-Nicolet, San Jose, CA, USA) spectrometer instrument.

## Supplementary Materials

Supplementary materials can be found at https://www.mdpi.com/1422-0067/21/11/3939/s1. Figure S1: photographs of PMMA mold (A), three-dimensional model (B) and stereomicroscope image (C) of the selected hole of PMMA mold, PDMS chip (1) with partially perforated holes fixed on a PMMA block (D), PDMS chip (2) with fully perforated holes fixed on a silicon wafer (E) and positioning device (F); Figure S2: schematic overview of PDMS chip fabrication procedures; Figure S3: photographs of hydrogel block mold and red ink experimental demonstration; Figure S4: SEM images of CaP structures observed at a3 and h3 in the pH gradient screening experiment through the platform; Figure S5: Fourier transform-infrared (FT-IR) spectra (A) and X-ray diffraction patterns (B) of CaP prepared in the scale-up experiments.

## Abbreviations

CaP	Calcium phosphates
HAp	Hydroxyapatite
Ca/P	Ca/P molar ratio
PDMS	Polydimethylsiloxane
PMMA	Polymethyl methacrylate
FE-SEM	Field emission scanning electron microscope
EP tube	Eppendorf tube
FT-IR	Fourier transform-infrared
XRD	X-ray diffraction
PCL	Polycaprolactone
