# Supplementary material for: A Microarray Screening Platform with an Experimental Conditions Gradient Generator for the High-Throughput Synthesis of Micro/Nanosized Calcium Phosphates
Source: Int J Mol Sci. 2020 May 30;21(11):3939. doi: 10.3390/ijms21113939 (PMC7312371; doi:10.3390/ijms21113939)
Supplement: Supplementary file 1 [file ijms-21-03939-s001.pdf]

International Journal of *Molecular Sciences*

Supplementary Materials

A Microarray Screening Platform with an Experimental Conditions Gradient Generator for the High-Throughput Synthesis of Micro/Nanosized Calcium Phosphates

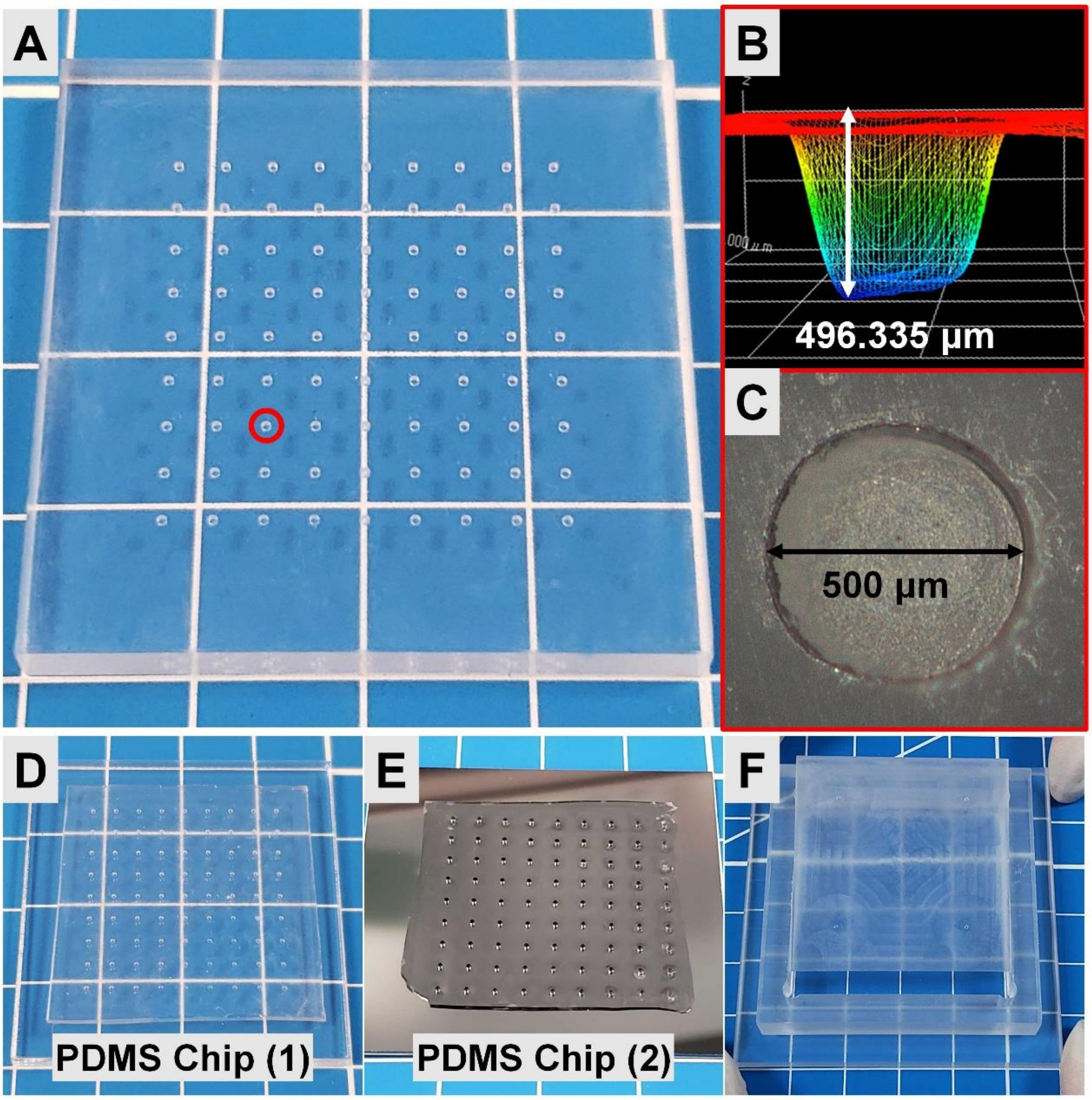

Figure S1. Photographs of PMMA mold (**A**), three-dimensional model (**B**) and stereomicroscope image (**C**) of the selected hole of PMMA mold, PDMS chip (1) with partially perforated holes fixed on a PMMA block (**D**), PDMS chip (2) with fully perforated holes fixed on a silicon wafer (**E**) and positioning device (**F**).

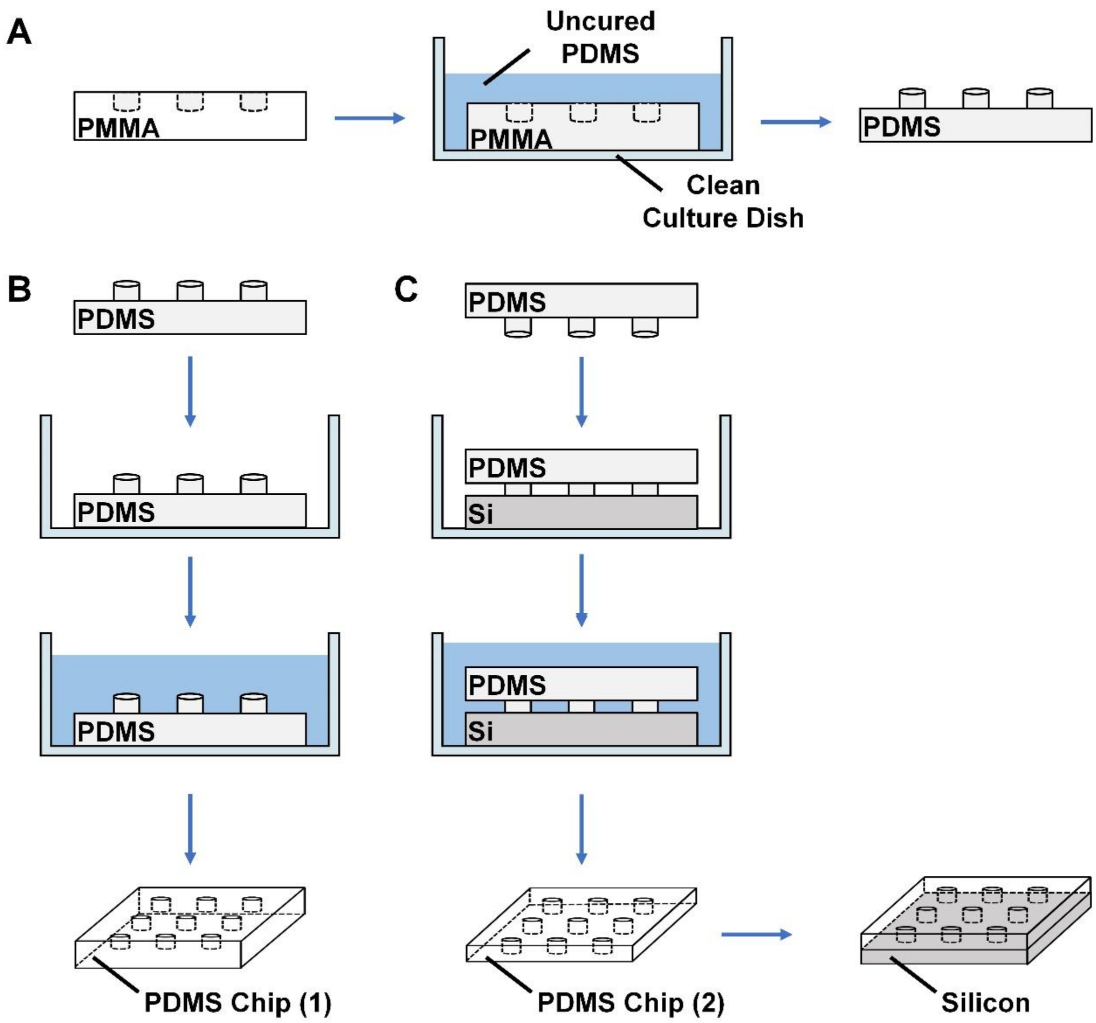

Figure S2. Schematic overview of PDMS chip fabrication procedures: (**A**) Fabrication of PDMS mold utilizing PMMA mold; (**B**) Fabrication PDMS chip (1) with partially perforated holes utilizing PDMS mold; (**C**) Fabrication PDMS chip (2) with fully perforated holes utilizing PDMS mold.

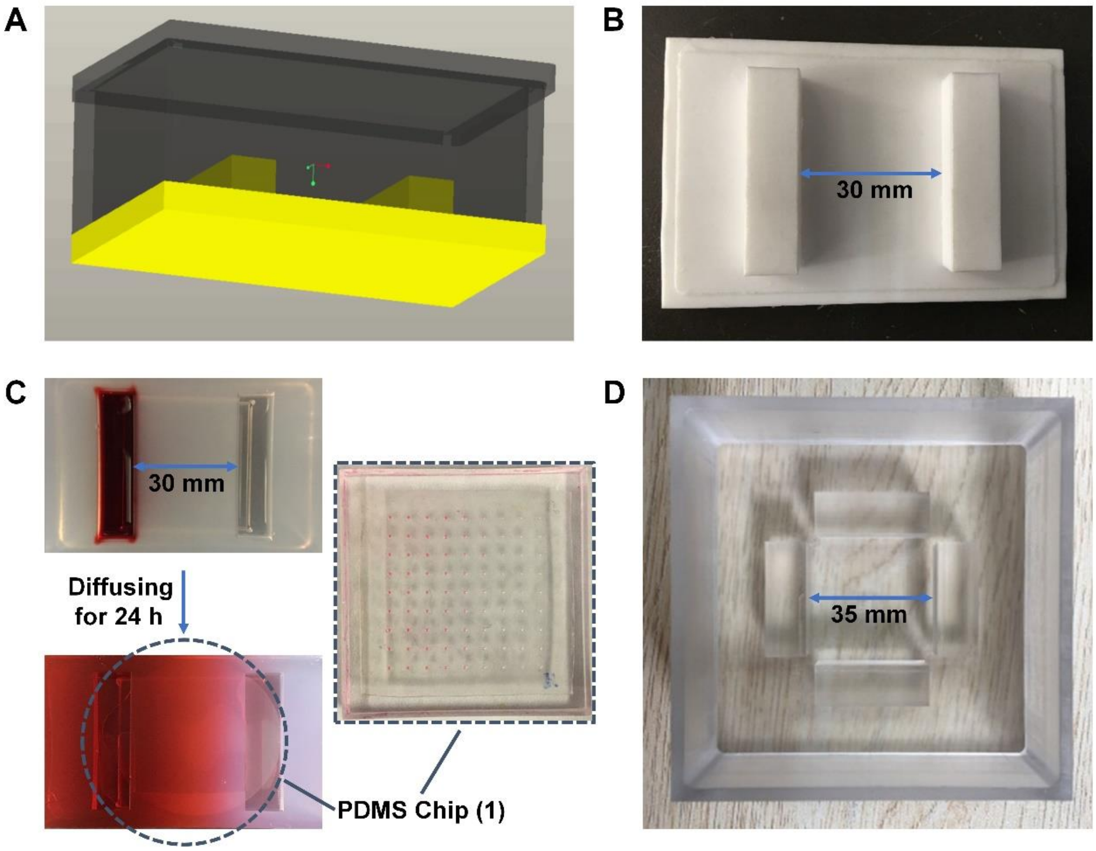

Figure S3. Photographs of hydrogel block mold and red ink experimental demonstration. Three-dimensional schematic of polycaprolactone (PCL) frame and Teflon base (**A**), photograph of Teflon base with two rectangular convex plates (**B**), red ink experiment for demonstration (**C**) and photograph of the PCL base four rectangular convex plates and the matched PCL frame (**D**).

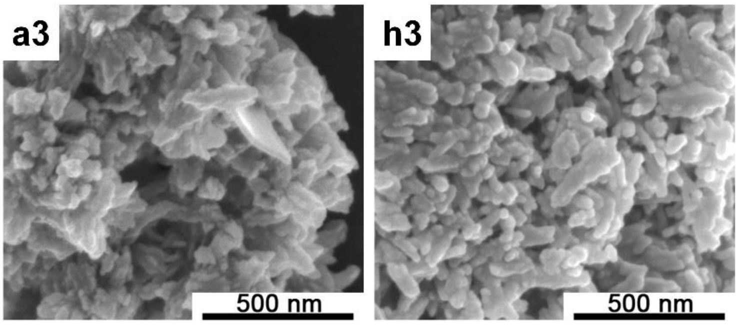

Figure S4. SEM images of CaP structures observed at a3 and h3 in the pH gradient screening experiment through the platform.

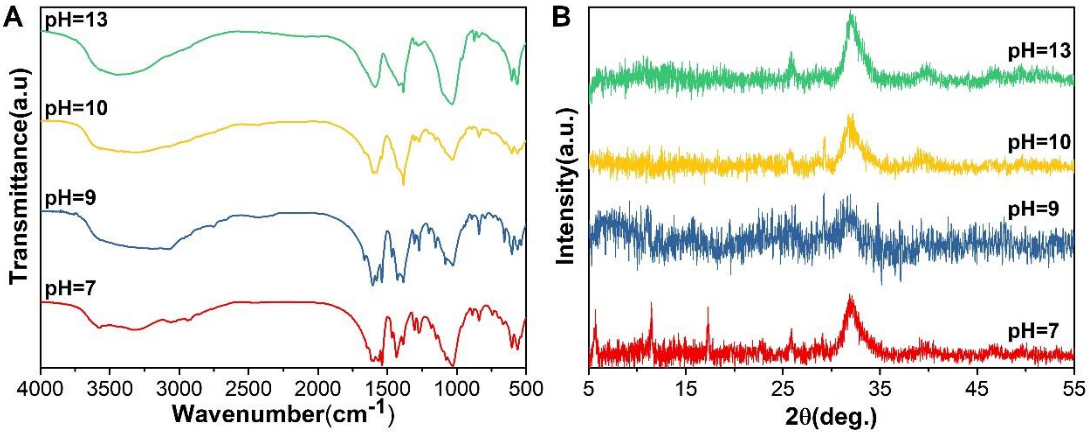

Figure S5. Fourier transform-infrared (FT-IR) spectra (**A**) and X-ray diffraction patterns (**B**) of CaP prepared in the scale-up experiments.
